# Oleaginous Microalga *Coccomyxa subellipsoidea* as a Highly Effective Cell Factory for CO_2_ Fixation and High-Protein Biomass Production by Optimal Supply of Inorganic Carbon and Nitrogen

**DOI:** 10.3389/fbioe.2022.921024

**Published:** 2022-06-06

**Authors:** Yu Liu, Dong Wei, Weining Chen

**Affiliations:** ^1^ School of Food Science and Engineering, Guangdong Province Key Laboratory for Green Processing of Natural Products and Product Safety, Engineering Research Center of Starch and Vegetable Protein Processing Ministry of Education, South China University of Technology, Guangzhou, China; ^2^ Research Institute for Food Nutrition and Human Health, Guangzhou, China; ^3^ School of Chemical and Biomedical Engineering, Nanyang Technological University, Singapore, Singapore

**Keywords:** oleaginous microalga, *Coccomyxa subellipsoidea*, CO_2_ fixation, high-protein biomass, photo-fermenter

## Abstract

Microalgae used for CO_2_ biofixation can effectively relieve CO_2_ emissions and produce high-value biomass to achieve “waste-to-treasure” bioconversion. However, the low CO_2_ fixation efficiency and the restricted application of biomass are currently bottlenecks, limiting the economic viability of CO_2_ biofixation by microalgae. To achieve high-efficient CO_2_ fixation and high-protein biomass production, the oleaginous microalga *Coccomyxa subellipsoidea* (*C. subellipsoidea*) was cultivated autotrophically through optimizing inorganic carbon and nitrogen supply. 0.42 g L^−1^ NaHCO_3_ supplemented with 2% CO_2_ as a hybrid carbon source resulted in high biomass concentration (3.89 g L^−1^) and productivity (318.33) with CO_2_ fixation rate 544.21 mg L^−1^ d^−1^ in shake flasks. Then, used in a 5-L photo-fermenter, the maximal protein content (60.93% DW) in batch 1, and the highest CO_2_ fixation rate (1043.95 mg L^−1^ d^−1^) with protein content (58.48% DW) in batch 2 of repeated fed-batch cultures were achieved under 2.5 g L^−1^ nitrate. The relative expression of key genes involved in photosynthesis, glycolysis, and protein synthesis showed significant upregulation. This study developed a promising approach for enhancing carbon allocation to protein synthesis in oleaginous microalga, facilitating the bioconversion of the fixed carbon into algal protein instead of oil in green manufacturing.

## 1 Introduction

Global climate change results from a massive emission of greenhouse gases (Blunden and Arndt, 2020; Pielke et al., 2022). Nowadays, CO_2_ concentration in the atmosphere has exceeded 400 ppm, mainly caused by the combustion of fossil fuels. CO_2_ biofixation by microalgae has obvious advantages on higher photosynthetic efficiency, faster cell growth, better environmental adaptability, and more high-value biomass coproduction than the higher plant and, thus, has been regarded as a promising approach for carbon capture and utilization (CCU) (Zhou et al., 2017; Cheng et al., 2019). However, the biotic (mainly biocontamination) and abiotic (water, weather, and material supply) factors leading to low algal biomass production are the bottlenecks restricting its application in the current agriculture-mode cultivation system of microalgae (Ruiz et al., 2016). Hence, enhancing the CO_2_ biofixation capacity of microalgae remains an urgent topic for sustainable biomass production and further value-added utilization.

Inorganic carbon source is the most critical factor for cell growth and CO_2_ fixation by autotrophic microalgae. Usually, dissolved CO_2_ and HCO_3_^−^ can be utilized as carbon sources, but low CO_2_ solubility limits the use for algal growth (Song et al., 2019b). To overcome this obstacle, bicarbonate can serve as a chemical absorbent to accelerate CO_2_ dissolution in an alkalic environment and is also a carbon source for microalgal growth (Song et al., 2019a), integrating the chemical absorption of CO_2_ with biofixation effectively. For example, the addition of 50 mM bicarbonate with 5% CO_2_ achieved a 27% increase in biomass productivity by *C. vulgaris* (Lohman et al., 2015). Hybrid CO_2_ capture based on bicarbonate to culture *Chlorella* sp. achieved a carbon conversion efficiency of over 60% (Song et al., 2019c). A 42% increase in cell growth of *Chlorella* sp. was achieved by supplying 5.0 g L^−1^ NaHCO_3_ to absorb CO_2_ (Tu et al., 2018). Hence, chemical absorption of CO_2_ coupled with biofixation as a hybrid CCU strategy is a promising approach for CO_2_ fixation and biomass production by microalgae.

Inorganic carbon assimilated *via* microalgae is further converted into various biochemical components including proteins, carbohydrates, lipids, and pigments. Recently, microalgae have been regarded as a novel protein source for meat analogs, which not only provides more nutritional ingredients compared to conventional plant-based protein but also reduces greenhouse gas emissions by replacing part of livestock breeding and farming (Fu et al., 2021). In addition to carbon sources, nitrogen is another core factor for microalgal growth, as a key element in amino acids, protein, and enzymes (Yadav et al., 2021). The nitrogen level in the medium directly affects nitrogen uptake (Wu and Miao, 2014) and the transcription of genes involved in nitrogen assimilation (Miller et al., 2010). A high level of nitrate can induce the high expression of genes related to nitrogen assimilation, such as nitrate reductase (NR) and nitrite reductase (NIR) (Chen et al., 2012). Abundant nitrate is transported into the cells and reduced to ammonium and further incorporated into α-oxoglutarate in the TCA cycle through the glutamine synthetase/glutamine oxoglutarate aminotransferase or glutamate synthase (GS/GOGAT) cycle for protein synthesis (Su, 2021). Additionally, replete nitrogen in the medium leads to nitrogen-rich algae cells with an intracellular C/N ratio of 6∼7 (Huang et al., 2018); thus, it is unnecessary to degrade the intra-nitrogen pool, including proteins, amino acids, and chlorophylls, for maintaining the cell growth. Conversely, the deprived nitrogen can result in an unbalanced intra-C/N ratio, and then the main nitrogen compounds are degraded to respond to the emergent demand for nitrogen. The previous study reported that the protein content of *Scenedesmus* sp. dramatically decreased by 70% (Pancha et al., 2014) and a 15% decline in *Chlamydomonas reinhardtii* (Dean et al., 2010) under nitrogen limitation, but the lipid content in these algae increased inversely. It suggested that maintaining a relatively high level of nitrogen in the medium can promote the photosynthetic carbon flow into protein synthesis by enhanced nitrogen assimilation.

As the first sequenced species of eukaryotic microalgae from the polar region, *C. subellipsoidea* is realized as an oleaginous microalga for biofuel production with the relatively fragile cell wall and many more genes involved in lipid biosynthesis (Blanc et al., 2012). Currently, most of the studies concerned the performance of *C. subellipsoidea* for lipid accumulation under nitrogen depletion, such as, high lipid content of 50.5% DW (Wang et al., 2017) and 52.16% DW under nitrogen limitation (Wang et al., 2019). Whereas lipid accumulation is generally achieved at the expense of cell growth under nitrogen deprivation (Corteggiani Carpinelli et al., 2014); additionally, *C. subellipsoidea* was only applied to biodiesel production, restricting its application potential in multiple areas, which further reduced the economic viability of *C. subellipsoidea* cultivation. In our previous study, a 2% CO_2_ supply could improve biomass productivity by encouraging autotrophic growth of *C. subellipsoidea* (Peng et al., 2016), suggesting the technical feasibility for CO_2_ fixation using *C. subellipsoidea*. However, so far, little is known about the potential application of this oleaginous microalga for enhancing CO_2_ fixation and protein coproduction by autotrophy simultaneously*.*

This study aimed to develop a hybrid CCU strategy for high-efficient CO_2_ fixation and high-protein biomass production through a combination of chemical absorption of CO_2_ and biofixation by autotrophic *C. subellipsoidea*. The hybrid carbon source optimized in shake flasks was further used in the batch and repeated fed-batch cultures with the optimized nitrogen supply in 5-L photo-fermenters. The expression of key genes involved in the central carbon and nitrogen metabolism was analyzed to reveal the regulation of enhanced CO_2_ fixation and carbon allocation to proteins synthesis in the cells.

## 2 Materials and Methods

### 2.1 Microalgae Strain and Seed Culture

The oleaginous microalga *C. subellipsoidea* was purchased from the Microbial Culture Collection of the National Institute for Environmental Studies (NIES) in Japan, with strain number NIES 2166. The seed culture was performed in 250-ml Erlenmeyer flasks containing 100 ml basal medium (Wang et al., 2019) with a modified concentration of NaNO_3_ (1.25 g L^−1^) and glucose (2.00 g L^−1^) as carbon source. The temperature was set at 25°C under continuous white LED of 80 μmol m^−2^ s^−1^ in a shaking incubator at 160 rpm.

### 2.2 Optimization of Inorganic Carbon Source in Shake Flasks

To explore the optimal inorganic carbon source for CO_2_ fixation, the autotrophic growth of *C. subellipsoidea* was conducted under the following conditions: 0.42 g L^−1^ NaHCO_3_ was added to the basal medium as the control. 2% CO_2_; 0.42, 0.63, and 0.84 g L^−1^ NaHCO_3_ with 2% CO_2_ supplementation were set up as the testing carbon source with 1.25 g L^−1^ NaNO_3_. The seed was inoculated into the 100 ml basal medium in 250-ml shake flasks with an initial cell density of about 1×10^7^ ml^−1^, and the other conditions were the same as the seed culture. The sample was taken every 2 days for measuring cell density, biomass concentration, and pH value. The nutrient composition, intracellular carbon and nitrogen contents in biomass, and dissolved inorganic carbon (DIC) in the medium were detected at the end of the cultures.

### 2.3 Batch and Repeated Fed-Batch Cultures in 5-L Photo-Fermenters

To verify the effect of the optimal carbon source obtained earlier, the batch culture was implemented in a 5-L photo-fermenter with automatic control of pH and temperature (25°C). The stock solution containing 8.4 g L^−1^ NaHCO_3_ was filtrated through a 0.22 μm membrane for removing bacteria and then added into the basal medium for a final concentration of 0.42 g L^−1^. Then, 2% CO_2_ was sparged continuously into the photo-fermenter controlled by a gas mass flow meter. The initial pH value was around 7.0 and then maintained at 8.0 by adding 0.5 M HCl solution during the incubation period. Four white LED panels were installed surrounding the glass fermenter to supply adjustable light intensity from 80 to 200 μmol m^−2^ s^−1^. The stirring speed was set from 150 to 300 rpm, and the aeration rate (1:1, *vvm*) was set at 4 L min^−1^ by pressed air. A gradient adjustment of light intensity and the stirring speed was set up with the increasing cell density. Especially, the initial NaNO_3_ concentration was increased to 2.5 g L^−1^ in the photo-fermenter to avoid fast depletion of nitrogen like 1.25 g L^−1^ NaNO_3_ in shake flasks.

To further enhance CO_2_ fixation, the repeated fed-batch cultures were carried out subsequently to relieve light limitation resulting from the deep green color caused by the high cell density in the batch culture. Batch 1 was performed in the basal medium containing 0.42 g L^−1^ NaHCO_3_ with 2% CO_2_ and 2.5 g L^−1^ NaNO_3_ for 6 days, and then 3 L culture liquor was pumped out and replaced by the same volume of the previous fresh basal medium, starting the batch 2 culture for another 7-days culture. The other culture conditions were the same as for the batch culture. The sample was taken to detect cell density every 24 h, and chlorophylls, intracellular carbon and nitrogen contents in biomass, and nitrate concentration in the medium were detected each 48 h. The nutrient composition in biomass was detected at the beginning and end of cultures.

### 2.4 Key Gene Expression Analysis for the Culture in 5-L Photo-Fermenters

To reveal the regulation of central carbon and nitrogen metabolism during autotrophic growth in 5-L photo-fermenters, the samples were taken at 192 h in the batch culture and at 48, 120, 144, 192, and 288 h in repeated fed-batch cultures for analysis of key genes’ expression levels, and then, the regulatory pathways were proposed.

### 2.5 Analytical Methods

#### 2.5.1 Cell Growth

Cell density was detected by CytoFLEX flow cytometry (Beckman-Coulter, United States); biomass dry weight (DW) was determined by the gravimetric method (Zhu et al., 2022); and specific growth rate, μ (d^−1^), was calculated according to the formula (Zhu et al., 2022).

#### 2.5.2 NO_3_^−^ Uptake and CO_2_ Fixation Rate

NO_3_^−^ concentration was determined using a multi-parameter water analyzer (HANNA, Italy) with the appropriative reagent. A total carbon analyzer (Vario TC, Elementar, Germany) was used to detect DIC in the medium. The intracellular carbon (Cc, % w/w) and nitrogen content (Nc, % w/w) were assayed using an element analyzer (Elementar, Germany), and then CO_2_ fixation rate (R_CO2_) and intracellular C/N ratio were calculated as follows:

$$R_{CO_{2}}\left(mg\;L^{-1}d^{-1}\right)=C_{C}P\frac{M_{CO_{2}}}{M_{C}},$$ (1)

$$Intra-C/Nratio=\frac{Cc/M_{C}}{Nc/M_{N}},$$ (2)

where *P* is the biomass productivity (mg L^−1^ d^−1^), M_C_ and M_N_ are the molecular weight of carbon and nitrogen, respectively, and M_CO2_ is the molecular weight of CO_2_.

#### 2.5.3 Nutrient Composition in Biomass

Protein content was determined by the Kjeldahl method (Chen and Vaidyanathan, 2013). Amino acid content and profile were analyzed according to the methods (Zhu et al., 2022) with modification. Briefly, 100 mg of lyophilized biomass powder was hydrolyzed with 5 ml of hydrochloric acid (6 mol L^−1^) in a water bath at 110°C for 24 h. The hydrolyzed sample was cooled to room temperature and filtered with a funnel and diluted to 25 ml. After that, 2 ml of the above sample was deacidified and then followed by the addition of 1 ml of 0.02 mol L^−1^ HCl as a buffer solution. Finally, 20 μl of the filtered sample was detected by an amino acid analyzer. Carbohydrate content was determined using the phenol-sulfuric acid method (Chen and Vaidyanathan, 2013). Total lipid content, fatty acids content, and profile were analyzed according to the previous study (Chen and Wei, 2015; Chen et al., 2021). Pigments were extracted by acetone (90%, v/v), and the qualitative and quantitative analysis of chlorophyll and carotenoids were carried out using the described method (Wang et al., 2020).

#### 2.5.4 Assay of Key Gene Expression Levels by qRT-PCR

Total RNA was extracted from fresh cells using TRIZOL (Invitrogen, Carlsbad, United States). RNA quality and quantity were evaluated by a micro-spectrophotometer (LabTech, Holliston, United States) (Supplementary Table S1). Evo M-MLV kit (Accurate Biotechnology, China) was employed to obtain cDNA, and qRT-PCR was performed on a real-time PCR detection system (Bio-rad, United States). The gene coding ribosomal protein L5 (*RibL5*) was used as the internal control (Peng et al., 2016). The primers (Supplementary Table S2) were designed by NCBI (https://www.ncbi.nlm.nih.gov/tools/primer-blast/), and the sequence of target genes was also searched in NCBI. The relative expression levels of target genes were normalized using *RibL5* as a reference gene by the 2^−ΔΔCt^ method (Peng et al., 2016).

### 2.6 Statistical Analysis

All data are presented as the means ± standard deviation (SD) from three replicates. Statistical analyses were carried out using Origin 9.0 software. The statistical significance was evaluated using a one-way analysis of variance (ANOVA) and LDS *t*-test with SPSS 26.0, and then, the significant levels were set at *p* < 0.05.

## 3 Results and Discussion

### 3.1 Effects of Inorganic Carbon Source on Cell Growth and CO_2_ Fixation in Shake Flasks

**FIGURE 1:**
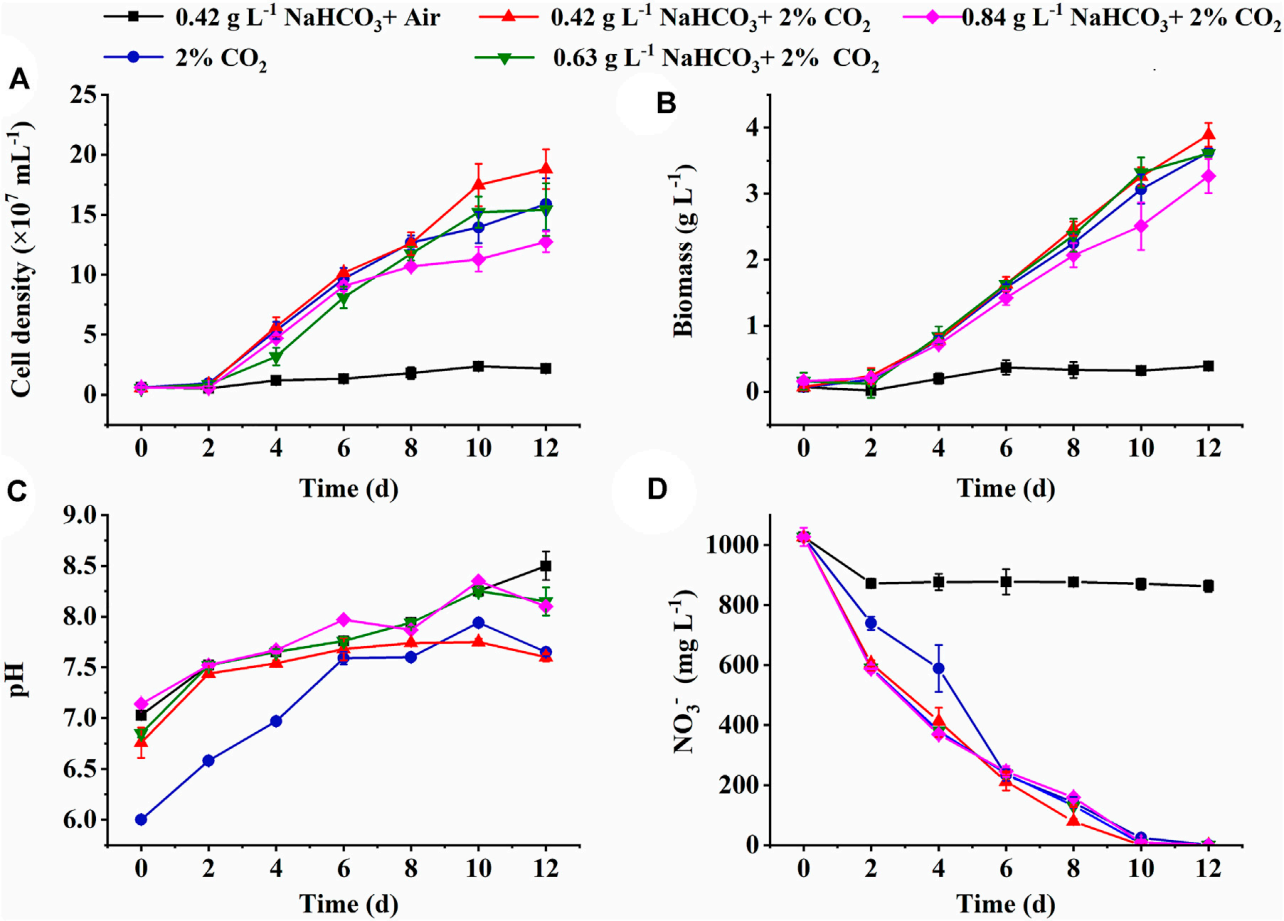
Effect of inorganic carbon source on the cell growth **(A)**, biomass production **(B)**, pH changes **(C)**, and NO_3_^−^ consumption **(D)** in the medium by autotrophic *C. subellipsoidea* in shake flasks.

The cell growth under various inorganic carbon sources is shown in Figure 1. The constant increase of cell density and biomass concentration was observed in all cultures supplemented with 2% CO_2_, much higher than the control (0.42 g L^−1^ NaHCO_3_ with air) (Figures 1A,B). The highest biomass yield (3.82 g L^−1^) and productivity (318.33 mg L^−1^ d^−1^) with final cell density (1.88 × 10^8^ ml^−1^) and R_CO2_ (544.21 mg L^−1^ d^−1^) were achieved under 0.42 g L^−1^ NaHCO_3_ with 2% CO_2_ (Table 1), 11.94-fold, 11.94-fold, 31.6-fold, and 13.3-fold higher than the control (*p* < 0.05), respectively. These results indicated that 0.42 g L^−1^ NaHCO_3_ with 2% CO_2_ was the optimal carbon source for biomass production and CO_2_ fixation, which was consistent with the previous reports that the highest biomass productivity (0.14 g L^−1^ d^−1^) of *C. vulgaris* was achieved under 5 mmol L^−1^ NaHCO_3_ with 5% CO_2_ as the carbon source (Lohman et al., 2015). More encouragingly, compared to the latest reports so far, R_CO2_ (544.21 mg L^−1^ d^−1^) obtained in the shake flasks was superior to that of 360.12 mg L^−1^ d^−1^ for *Chlorella fusca* (Zhu et al., 2020), 350.0 mg L^−1^ d^−1^ for *Scenedesmus* sp. (Rodas-Zuluaga et al., 2021), 470.0 mg L^−1^ d^−1^ for *Chlorella* sp. (Yadav et al., 2020), and 350.0 mg L^−1^ d^−1^ for *N. oceanica* (Wang et al., 2022) by autotrophic culture in shake flasks. These results might be attributed to the sufficient DIC supplied by CO_2_ and NaHCO_3_ as the hybrid carbon sources in the medium for photosynthesis. It was observed that the DIC concentration (51∼79 mg L^−1^) under hybrid carbon sources was significantly higher than that of around 10 mg L^−1^ under 0.42 g L^−1^ NaHCO_3_ and 41 mg L^−1^ under 2% CO_2_, respectively, but the excess DIC under 0.63 and 0.84 g L^−1^ NaHCO_3_ with 2% CO_2_ supplement was shown to decrease the biomass yield and R_CO2_ (Table 1).

**TABLE 1:** Biomass production, CO_2_ photosynthetic fixation, and nitrate uptake by autotrophic *C. subellipsoidea* under various inorganic carbon sources in shake flasks.

Variable	Inorganic carbon source				
	0.42 g L^−1^ NaHCO_3_ + air	2% CO_2_	0.42 g L^−1^ NaHCO_3_ + 2% CO_2_	0.63 g L^−1^ NaHCO_3_ + 2% CO_2_	0.84 g L^−1^ NaHCO_3_ + 2% CO_2_
Biomass production					
Yield (g L^−1^)	0.32 ± 0.06^e^	3.43 ± 0.26^c^	3.82 ± 0.18^ab^	3.61 ± 0.26^bc^	3.11 ± 0.2^d^
Productivity (mg L^−1^ d^−1^)	26.67 ± 0.50^e^	285.83 ± 5.67^c^	318.33 ± 1.52^ab^	300.83 ± 2.16^bc^	259.17 ± 16.67^d^
CO2 fixation					
Intracellular carbon (% DW)	45.64 ± 0.56^b^	48.58 ± 0.03^a^	48.77 ± 0.15^a^	48.60 ± 0.21^a^	48.61 ± 0.04^a^
Extracellular DIC (mg L^−1^)	9.69 ± 0.57^e^	41.40 ± 1.90^d^	51.00 ± 1.93^c^	58.58 ± 0.88^b^	79.16 ± 0.37^a^
R_CO2_ (mg L^−1^ d^−1^)	40.91 ± 0.50^e^	523.10 ± 0.55^b^	544.21 ± 0.17^a^	515.51 ± 0.30^c^	443.67 ± 1.70^d^
Nitrate uptake					
NO_3_^−^ uptake rate (mg L^−1^ d^−1^)	13.63 ± 2.03^c^	100.25 ± 2.56^b^	118.50 ± 1.59^a^	102.18 ± 3.15^b^	101.75 ± 2.50^b^
Intracellular nitrogen (% DW)	6.21 ± 0.33^b^	5.51 ± 0.26^b^	5.34 ± 0.11^c^	6.57 ± 0.09^a^	6.12 ± 0.03^b^
Intracellular C/N ratio	7.78 ± 0.01^d^	10.31 ± 0.50^b^	10.66 ± 0.50^a^	9.14 ± 0.12^c^	9.27 ± 0.19^c^

Different letters in superscripts within one row present a significant difference (*p* < 0.05). Letters denoted the differentiation among different inorganic carbon sources.

In addition to DIC, the most stable pH in the medium was also observed under the optimal carbon source (0.42 g L^−1^ NaHCO_3_ and 2% CO_2_) with the minimum pH fluctuation at 1.15, compared to 1.47 in the control and around 1.94 in the other two cultures (Figure 1C). It was reported that bubbled CO_2_ captured by the hybrid carbon source (NaHCO_3_ and CO_2_) and stored as HCO_3_^−^ could drop the pH value in the medium and weaken the effect of pH rise resulting in nitrate and HCO_3_^−^ consumption *via* algal cell growth (Wang et al., 2018; Zhu et al., 2020). When HCO_3_^−^ is utilized, OH^−^ can be released and then recycled to capture subsequent CO_2_ to form bicarbonate again (Gardner et al., 2012). The advantage of the optimal carbon source provided an effective buffer system for steady pH and sufficient DIC for photosynthesis.

It is also noticed that nitrate almost ran out on the 8^th^ day in the group of hybrid carbon sources (Figure 1D), where NO_3_^−^ uptake rate was significantly higher than that of the single carbon sources (0.42 g L^−1^ NaHCO_3_ or 2% CO_2_). The highest NO_3_^−^ uptake rate (118.50 mg L^−1^ d^−1^) under the optimal carbon source was 8.7-fold higher than that of the control (13.63 mg L^−1^ d^−1^) (Table 1) due to the rapid cell growth. It resulted in the N-depletion, and then the low nitrogen with high carbon content intracellularly led to the unbalanced C/N ratio of 9 ∼ 11, which exceeded the normal range of 6–7 in algae cells (Huang et al., 2018), under hybrid carbon sources or 2% CO_2_ (Table 1); thus, the slow increase of cell density and biomass accumulation from the 8^th^ day to the end of culture might be caused by the N-depletion (Figures 1A,B). The results suggested that sufficient nitrogen is critical to maintain the balanced C/N ratio intracellularly for fast cell growth and CO_2_ fixation. Thus, the nitrate concentrate should be elevated in the following experiments.

### 3.2 Cell Growth and CO_2_ Fixation in the Batch and Repeated Fed-Batch Cultures in 5-L Photo-Fermenters

**FIGURE 2:**
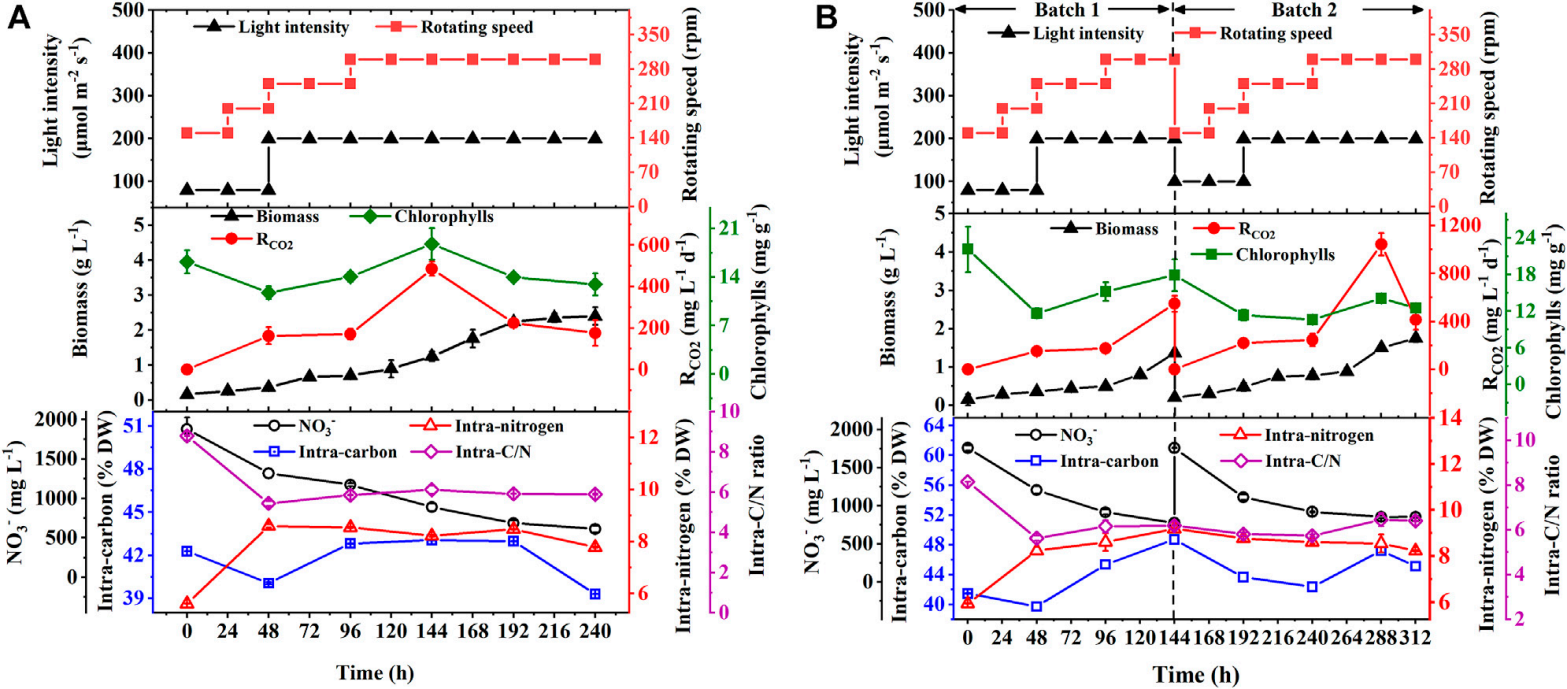
Biomass production, chlorophyll contents and CO_2_ fixation rate (R_CO2_), NO_3_^−^ consumption, and the intracellular contents of carbon and nitrogen with their ratios in biomass during the batch **(A)** and repeated fed-batch cultures **(B)** of autotrophic *C. subellipsoidea* in 5-L photo-fermenters under the controllable operation parameters.

To verify the effect of the optimal hybrid carbon source and optimize nitrate concentrations in a scale-up culture, the batch and repeated fed-batch cultures were conducted in 5-L photo-fermenters. In Figure 2A, a slow increase of biomass concentration within the first 48 h in the batch culture was observed, and then it increased constantly till the stationary phase, along with biomass yield (1.75 g L^−1^) and productivity (175.00 mg L^−1^ d^−1^) (Table 2). The long stationary phase lasted from 192 h to the end of the culture; this might be due to the limitation of effective depth of light penetration (Pilon et al., 2011), resulting in dark green color by increasing cell density. Although the gradient adjustment of light intensity and stirring speed could improve the mixing and reduce the light-shading effect to some extent, the algal cells still lack adequate light required by photosynthesis. It implies that the well-distributed light field inside a photobioreactor is still the key challenge for the larger-scale cultivation of microalgae (Sun et al., 2016). The intracellular chlorophylls, carbon content, and R_CO2_ increased at the exponential phase and then decreased synchronously, and the average R_CO2_ (252.18 mg L^−1^ d^−1^) was obtained along with the intracellular carbon (39.3% DW) and chlorophylls content (12.9 mg g^−1^). It was noticed that the content of intracellular substances, like chlorophylls, carbon, and nitrogen showed a strong fluctuation in the first 48 h (Figure 2A). It might be ascribed that microalgal cells required new homeostasis established *via* metabolic regulation when it was transferred from the shake flask to the photo-fermenter or fresh medium was added into the culture (Moejes et al., 2017). Regarding nitrogen assimilation, it was found that NO_3_^−^ concentration constantly decreased with uptake rate (126.56 mg L^−1^ d^−1^) and remained at 610.90 mg L^−1^ at the end of culture under 2.5 g L ^−1^ NaNO_3_ (Table 2). The intracellular nitrogen content and C/N ratio were kept stable at around 8% DW and six, respectively, from 48 h to the end of the culture (Figure 2A). The sufficient nitrate in the medium and balanced intracellular C/N ratio suggested that the slow increase of biomass concentration and the decline of R_CO2_ after 144 h were caused by the light attenuation instead of the decreased nitrogen.

**TABLE 2:** Biomass production, CO_2_ fixation, and nitrate uptake by autotrophic *C. subellipsoidea* during the batch and repeated fed-batch cultures under 2.5 g L^−1^ NaNO_3_ in 5-L photo-fermenters.

Variable	Batch culture	Repeated fed-batch culture	
		Batch 1	Batch 2
Biomass production			
*μ* (d^−1^)	0.25 ± 0.02^b^	0.30 ± 0.01^b^	0.37 ± 0.02^a^
Yield (g L^−1^)	1.75 ± 0.11	1.23 ± 0.04	1.54 ± 0.01
Productivity (mg L^−1^ d^−1^)	175.00 ± 9.00^c^	204.17 ± 5.67^b^	220.28 ± 1.43^a^
CO2 fixation and nitrate uptake rate			
Ave. R_CO2_ (mg L^−1^ d^−1^)	252.18 ± 3.54^b^	364.57 ± 1.68^a^	364.43 ± 0.57^a^
Max. R_CO2_ (mg L^−1^ d^−1^)	484.93 ± 4.98^c^	848.19 ± 25.67^b^	1043.95 ± 72.01^a^
NO_3_^−^ uptake rate (mg L^−1^ d^−1^)	126.56 ± 6.82^b^	168.75 ± 10.83^a^	128.78 ± 4.85^b^
Final concentration of NO_3_^−^ (mg L^−1^)	610.90 ± 39.88^b^	774.75 ± 18.03^a^	855.00 ± 12.73^a^

The significant difference of p < 0.05 is presented between the batch and repeated fed-batch cultures.

To overcome the drawback, repeated fed-batch culture was adopted to weaken light attenuation by renewing the culture liquor through harvesting biomass and replacing fresh medium. The biomass concentration and R_CO2_ constantly increased to 1.37 g L^−1^ and 548.19 mg L^−1^ d^−1^ in batch 1, respectively, and then an exponential phase of cell growth was observed again in batch 2 (Figure 2B). As summarized in Table 2, the specific growth rate (0.30 d^−1^ and 0.37 d^−1^), biomass productivity (204.17 mg L^−1^ d^−1^ and 220.28 mg L^−1^ d^−1^), and average R_CO2_ (364.57 mg L^−1^ d^−1^ and 364.43 mg L^−1^ d^−1^) in batch 1 and batch 2 of repeated fed-batch culture were significantly 1.2-∼1.5-fold, 1.2-∼1.3-fold, and 1.5-fold higher than those of the batch culture in 5-L photo-fermenters, respectively (*p* < 0.05). Especially, the maximum R_CO2_ (1043.95 mg L^−1^ d^−1^) in batch 2 was 2.2-fold higher than that of 484.93 mg L^−1^ d^−1^ in the batch culture (*p* < 0.05). The enhanced biomass production and CO_2_ fixation in repeated fed-batch culture indicated that biomass harvesting and medium renewing could effectively relieve lighting shade. The previous study also reported that maintaining suitable cell density through biomass harvesting could provide abundant light for photophosphorylation, which further produced more assimilatory power (ATP and NADPH) for CO_2_ fixation (Naduthodi et al., 2021). Additionally, R_CO2_, intracellular chlorophylls, and carbon content in the two batch cultures also exhibited synchronized change trends in the exponential phase. Notably, those in batch 2 sharply decreased at 288 h, which might be a photoprotective mechanism against photodamage caused by intense photosynthesis *via* reducing chlorophyll content to slow down CO_2_ fixation (Humby et al., 2009). In case of nitrogen assimilation, NO_3_^−^ concentration in the medium was always kept above 775 mg L^−1^ with a NO_3_^−^ uptake rate of 168.75 mg L^−1^ d^−1^ (batch 1) and 128.78 mg L^−1^ d^−1^ (batch 2), which were higher than those of the batch culture (Table 2); thereby the higher intracellular nitrogen contents (8.2∼9.2% DW) and balanced C/N ratio around 6.0 were achieved (Figure 2B). These results suggested that compared to 1.25 g L^−1^ NaNO_3_ in shake flasks, 2.5 g L^−1^ NaNO_3_ in 5-L photo-fermenter eliminated the N-limitation, leading to a balanced intracellular C/N ratio. Taken together, repeated fed-batch culture under higher nitrate would be a favorable option for scale-up application in CO_2_ fixation, and the renewal rate of the medium should be optimized for further improvement.

### 3.3 Nutrient Composition of Biomass in a Shake Flask and 5-L Photo-Fermenter

**FIGURE 3:**
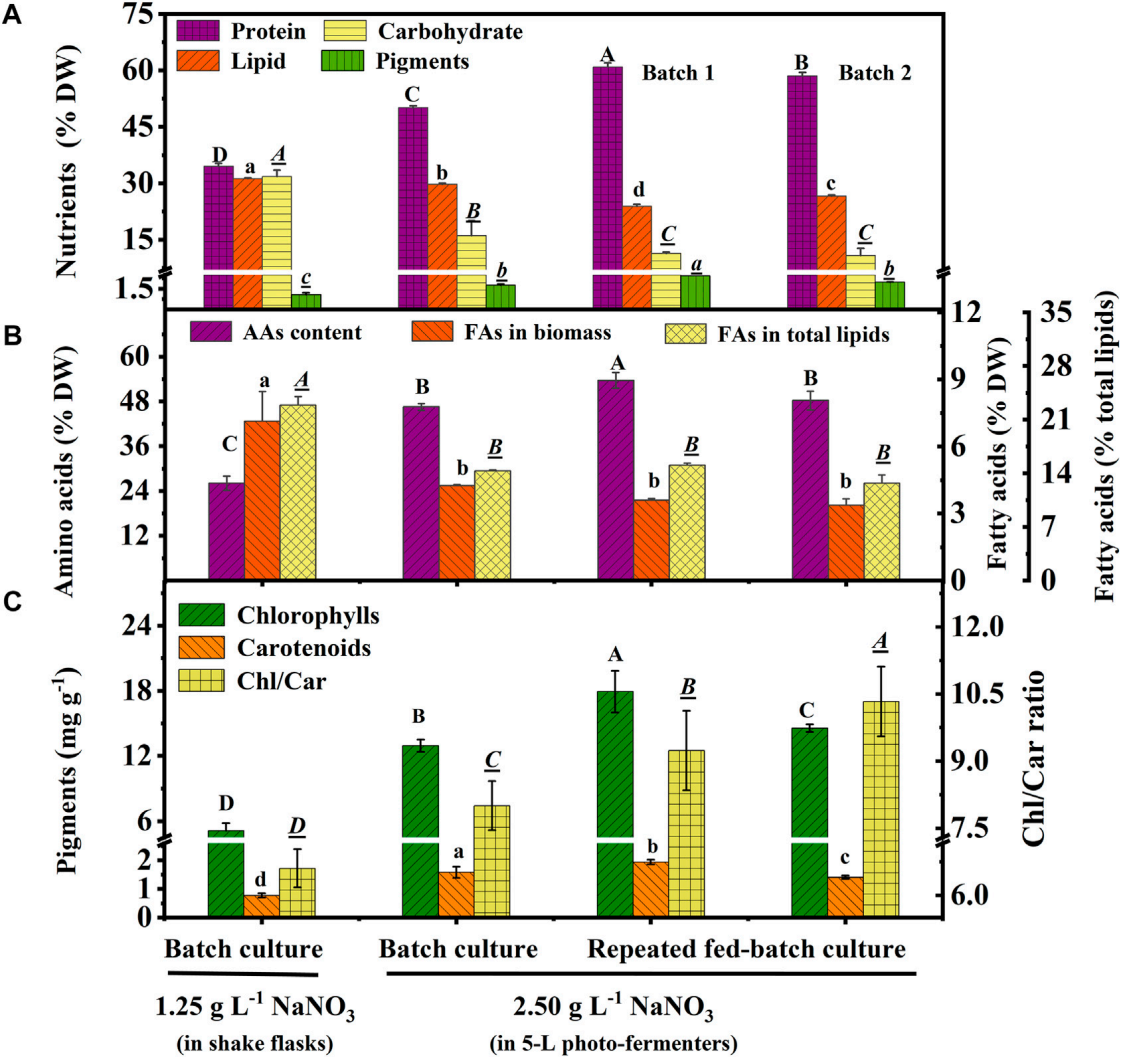
Nutrients **(A)**, amino acids and fatty acids **(B)**, and pigment contents with the ratio **(C)** in biomass of autotrophic *C. subellipsoidea* in the batch and repeated fed-batch cultures under various nitrate concentrations in shake flasks and 5-L photo-fermenters. The different letters on the bar represent the significant difference (*p* < 0.05) between the groups.

Protein, carbohydrate, lipid, and pigments are the main nutritional components in biomass. As shown in Figure 3A, protein content increased to 50.1% DW in the batch culture 60.93% (batch 1) and 58.48% DW (batch 2) in repeated fed-batch culture under 2.5 g L^−1^ NaNO_3_, which was 45.18%, 76.56%, and 69.46% higher than that of 34.51% DW under 1.25 g L^−1^ NaNO_3_ under the optimal carbon source in shake flasks. The pigment content (1.45% DW) of the batch culture in the 5-L photo-fermenter showed a 150% increase compared to that of 0.58% DW in shake flasks; while, 2.43% DW (batch 1) and 1.96% DW (batch 2) of repeated fed-batch culture in 5-L photo-fermenter were also achieved, 4.19-fold and 3.43-fold higher than that in shake flasks, respectively (*p* < 0.05). Contrarily, the culture in shake flasks resulted in higher contents of carbohydrate (31.81% DW) and lipid (31.17% DW), 1.1-∼1.3-fold and 2.0-∼3.0-fold higher than those (10.77∼16.06% DW, 23.89∼29.75% DW) in photo-fermenters (*p* < 0.05). The nitrogen level is known to alter the carbon flux, and the high level of nitrogen could accelerate protein and chlorophyll synthesis, but the limited nitrogen induced lipid synthesis (Zhu et al., 2022). It was observed that the nitrate was completely depleted on the 10^th^ day with low intracellular nitrogen content (5.34% DW) and higher intracellular C/N ratio (10.66) in shake flasks compared to that in 5-L photo-fermenters (Figure 1D and Table 1). The low content of protein and pigments in shake flasks indicated that these nitrogenous compounds might be degraded to maintain normal metabolism under N limitation (Wu and Miao, 2014). Contrarily, in 5-L photo-fermenters, the intracellular nitrogen content (8%∼9% DW) and C/N ratio (6.0) were maintained at a suitable level, enhancing carbon allocation to protein and pigment synthesis. Attractively, the high-protein content (>60% DW) was achieved in repeated fed-batch culture (Figure 3A), significantly higher than that of 57.36% DW for *Chlorella* sp. (UKM2), 48.6% DW for *Scenedesmus* sp. (UKM9) (Hariz et al., 2019), and 52.3% DW for *Chlorella fusca* (Duarte et al., 2016) under autotrophic cultivation for CO_2_ fixation. It is suggested that the high-protein biomass of *C. subellipsoidea* can be expanded to the application of algae-based protein for meat analogous and animal feed (Fu et al., 2021).

Amino acids (AAs) are the critical compounds of cell nutrition. In Figure 3B, the changing trend of AA content under different culture conditions was coincident with that of protein. The highest content of AAs in 5-L photo-fermenters was 53.65% DW, 2.1-fold higher than that in shake flasks. The notable difference in AA contents might also be due to the distinct nitrate level described earlier. As for the profile of AAs shown in Table 3, the biomass contained 18 types of AAs, including eight types of essential amino acids (EAAs) for humans. Especially, the high contents of EAAs (16.12%–18.96% DW) were found in biomass from repeated fed-batch culture, leading to attractive application in healthy foods. Additionally, the typical protein diets in animal feed production require supplementation with EAAs, such as lysine (Lys), methionine (Met), threonine (Thr), and tryptophan (Trp) (Sun et al., 2020). The highest contents of these limiting EAAs reached 3.44, 0.86, 2.57, and 0.04% DW in batch 1 of repeated fed-batch culture, respectively, similar to that of soybean and corn (Lum et al., 2013). Consequently, the biomass produced by repeated fed-batch culture could be a good feedstock for animal feed production in the future (Sun et al., 2020).

**TABLE 3:** Amino acid and fatty acid profiles in the finally harvested biomass of autotrophic *C. subellipsoidea* under various nitrate concentrations.

Variable	1.25 g L^−1^ NaNO_3_ (in shake flasks)	2.5 g L^−1^ NaNO_3_ (in 5-L photo-fermenters)		
	Batch culture	Batch culture	Repeated fed-batch culture	
			Batch 1	Batch 2
EAAs (% DW)				
Lys	1.95 ± 0.26	3.00 ± 0.11	3.44 ± 0.43	2.77 ± 0.58
Trp	0.03 ± 0.00	0.04 ± 0.01	0.04 ± 0.01	0.04 ± 0.00
Phe	1.55 ± 0.21	2.39 ± 0.09	2.80 ± 0.44	2.36 ± 0.21
Met	0.39 ± 0.08	0.76 ± 0.10	0.86 ± 0.04	0.77 ± 0.10
Thr	1.39 ± 0.23	2.22 ± 0.16	2.57 ± 0.71	2.43 ± 0.80
Ile	1.02 ± 0.02	1.83 ± 0.02	2.11 ± 0.12	1.70 ± 0.20
Leu	2.11 ± 0.23	3.64 ± 0.28	4.26 ± 0.28	3.64 ± 0.12
Val	1.51 ± 0.33	2.51 ± 0.52	2.88 ± 0.06	2.41 ± 0.05
Total EAA	9.96	16.40	18.96	16.12
NEAAs (% DW)				
His	0.67 ± 0.04	0.92 ± 0.21	1.05 ± 0.25	1.41 ± 0.02
Asp	2.76 ± 0.12	4.53 ± 0.23	5.41 ± 0.56	4.31 ± 0.52
Ser	1.39 ± 0.22	1.96 ± 0.21	2.26 ± 0.70	1.99 ± 0.21
Glu	3.43 ± 0.12	6.34 ± 0.02	7.67 ± 0.66	7.09 ± 0.11
Gly	1.65 ± 0.17	2.61 ± 0.08	2.96 ± 0.37	2.47 ± 0.33
Ala	2.25 ± 0.99	3.87 ± 0.06	4.44 ± 0.98	3.70 ± 0.67
Cys	0.10 ± 0.07	0.18 ± 0.04	0.21 ± 0.07	0.46 ± 0.01
Tyr	0.98 ± 0.06	1.77 ± 0.02	2.06 ± 0.23	2.09 ± 0.17
Arg	1.94 ± 0.22	6.10 ± 0.34	6.53 ± 0.15	6.09 ± 0.42
Pro	1.05 ± 0.01	1.91 ± 0.12	2.07 ± 0.12	2.51 ± 0.21
Total NEAAs	16.14	30.12	34.68	32.11
Fatty acids (% TFAs)				
C16:0	21.89 ± 0.13	22.31 ± 0.60	24.99 ± 0.91	26.34 ± 0.56
C18:1 (∆9)	22.90 ± 0.43	3.59 ± 0.57	ND	ND
C18:2 (∆9, 12)	26.71 ± 0.23	23.74 ± 0.16	25.83 ± 1.21	24.23 ± 0.52
C18:3 (∆9, 12, 15)	22.95 ± 0.28	33.73 ± 2.17	35.60 ± 1.73	32.78 ± 0.73
C20:2 (∆11, 14)	ND	1.94 ± 0.07	2.86 ± 0.45	2.49 ± 0.35
C20:3 (∆11, 14, 17)	5.55 ± 0.05	10.68 ± 0.43	10.71 ± 0.19	9.22 ± 0.31
DLU	1.64	2.23	1.96	1.78
ω-6/ω-3	1.16	0.70	0.73	0.74

EAAs, essential amino acids for humans; NEAAs, non-essential amino acids; DLU, degree of lipid unsaturation; ND, not detected.

Fatty acid content and percentage in total lipids are shown in Figure 3B, and the profile is shown in Table 3. Total fatty acid (TFA) contents were around 3∼4% DW and 12.0 ∼15.0% of total lipids in 5-L photo-fermenters, significantly lower than that of 7.13% DW and 22.88% of total lipids in shake flasks (*p* < 0.05). The decline of TFA content could also be ascribed that a high concentration of nitrate favored for protein and chlorophylls but not for fatty acids (Gao et al., 2018). In Table 3, the profile of fatty acids consisted of both C16 to C18 with over 85% of TFAs, which satisfied the biodiesel quality (Wu and Miao, 2014) in all cultures. The polyunsaturated fatty acids (PUFAs) accounted for 75% of TFAs in batch 1 of repeated fed-batch culture, 1.4-fold higher than that in shake flasks. The monounsaturated fatty acids of C18:1 sharply decreased from over 22.0% to 4.0% of TFAs or less and were transformed into the PUFAs (C18:3 and C20:3) under increased nitrate in 5-L photo-fermenter. It was the opposite that the production of PUFAs (C18:2 and C18:3) in *C. vulgaris* shifted to the saturated or monounsaturated fatty acids (C18:0 and C18:1) production under N limitation (Griffiths et al., 2014). This suggested that sufficient nitrogen contributed to the synthesis of PUFAs, leading to a higher degree of lipid unsaturation (DLU) compared to the limited nitrogen (Table 3). Additionally, the ω-6/ω-3 ratio, being essential for human health at the ideal ratio of 1:1 (Jo et al., 2021), was from 0.70 to 1.16 in all cultures (Table 3), which all got close to this ideal ratio. In this case, the composition of fatty acids in the biomass of *C. subellipsoidea* satisfied with nutrient intake for human health.

In terms of composition of pigments (Figure 3C), high chlorophyll (Chl) content (12.9∼17.9 mg g^−1^) and a significant increase of carotenoid (Car) content (1.4∼1.9 mg g^−1^) were observed in the 5-L photo-fermenter; otherwise, only 5.11 and 0.77 mg g^−1^ for Chl and Car contents with an obvious decline in the Chl/Car ratio from 8.0∼10.3 to 6.6 in shake flasks, suggesting a relative increase of Car content under the stress of depleted nitrogen (Simionato et al., 2013). It implied that maintaining a high level of nitrate in the medium was essential for pigment synthesis. Taken together, the high-protein biomass of *C. subellipsoidea* in the present work has great potential application as feedstock for high-quality food ingredients, healthy foods, and animal feed production in the future.

### 3.4 Key Gene Expression Analysis in the Batch and Repeated Fed-Batch Cultures in 5-L Photo-Fermenters

**FIGURE 4:**
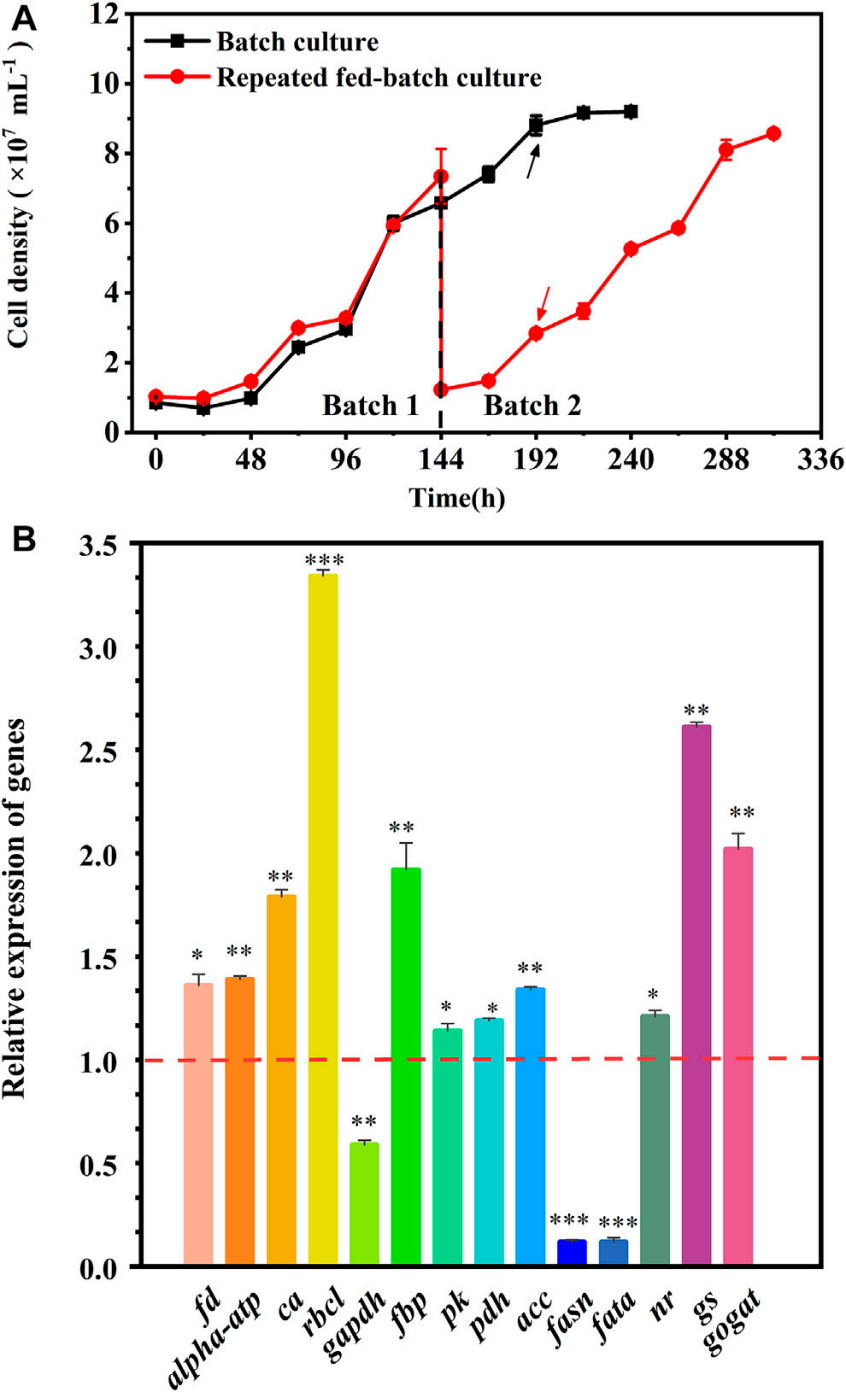
Cell growth **(A)** and the relative expression of key genes **(B)** in the batch and repeated fed-batch cultures in 5-L photo-fermenters. The expression levels of key genes encoding key enzymes in the batch culture at 192 h are set to 1.0 as the control. The value > 1.0 means the upregulation of key genes, while the value <1.0 means the downregulation of key genes. Significant difference is presented at *p < 0.05, **p < 0.01, and ***p < 0.001 when compared with the control, respectively.

The cell growth in the batch culture reached the stationary phase at 192 h, but a rapid growth was observed in batch 2 of repeated fed-batch culture due to the weakened light-shading (Figure 4A). Hence, to explore the regulation of cells’ response to the variation, the fresh cells at 192 h in the batch culture and batch 2 of repeated fed-batch cultures were collected to analyze key genes’ expression. As shown in Figure 4B, compared to that in the batch culture, the expression of ferredoxin (*fd*) and chloroplast ATP synthase (*alpha-atp*) in batch 2 was 1.36-fold and 1.39-fold upregulated, which might produce more ATP and NADPH to promote CO_2_ fixation (Naduthodi et al., 2021). The expression of *rbcl* coding ribulose-1,5-bisphosphatecarboxylase/oxygenase (RuBisCo), the critical enzyme catalyzing CO_2_ fixing, was 3.34-fold upregulated, which might convert more CO_2_ into glycerate-3P for cell growth (Peng et al., 2016; Li et al., 2021). It was reported that overexpression of *rbcl* in oleaginous microalga dramatically accelerated CO_2_ fixation, leading to a 46% increase in biomass yield (Wei et al., 2017). The transcription of *ca* encoding carbonic anhydrase (CA) was upregulated by 1.79-fold, which perhaps provided more CO_2_ for RuBisCo (Wang et al., 2015). The expression of fructose bisphosphatealdolase (*FBP*) in chloroplast was 1.92-fold upregulated, which might enhance the conversion of glyceraldehyde-3-phosphate (G3P) into fructose-1,6-biphosphate; and the overexpression of *fbp* was previously reported to enhance photosynthesis and growth rate in *Synechococcus* sp. PCC 7002 (De Porcellinis et al., 2018). But the transcription of gene coding glyceraldehyde-3-phosphate dehydrogenase (GAPDH), the key enzyme for G3P synthesis, was 0.59-fold downregulated, which did not match up with the enhanced CO_2_ fixation in batch 2. The genes’ expression changed rapidly, and the inconsistent transcription of *gapdh* at 192 h of repeated fed-batch culture might be affected by changing environment in the medium. Overall, the upregulated gene expression in photosynthesis assuredly enhanced CO_2_ fixation in batch 2. The enhanced CO_2_ fixation could provide more G3P for glycolysis, where the gene encoding pyruvate kinase (PK) was upregulated, leading to produce more ATP and pyruvate; and the gene encoding pyruvate dehydrogenase complex (PDH) was dramatically 1.19-fold upregulated; the upregulation of *pk* and *pdh* rapidly converted pyruvate into acetyl-CoA, which widely participates in TCA cycle, amino acids synthesis, and lipid metabolism (Li et al., 2021). Ordinarily, when the carbon source is no longer the limiting factor, a high level of nitrogen has a positive effect on cell growth and protein synthesis (Pancha et al., 2014). In this work, the final level of nitrate in batch 2 was 1.4-fold higher than that in the batch culture (Table 2); the replete nitrate might induce upregulation of genes involved in nitrogen assimilation. Consequently, the gene encoding nitrate reductase (NR) was notably upregulated to assimilate extracellular nitrogen into ammonium in cells. The gene encoding glutamine synthetase (GS), assimilating ammonium into carbon compounds in a nitrogen-rich environment, was also notably upregulated. Upregulation of the GS/GOGAT pathway promoted the integration of the carbon skeleton from the TCA cycle and assimilated nitrogen for protein synthesis. The upregulation of gene coding acetyl-CoA carboxylase (ACC) in lipid synthesis was observed, which provided an abundant substrate of malonyl-CoA for fatty acid synthesis; but the genes encoding fatty acid synthase (FASN) and fatty acyl-ACP thioesterase A (FATA), key enzymes involving in the acyl chain elongation for long-chain fatty acids, were both significantly downregulated by 0.12-fold in batch 2, which prevented the synthesis of fatty acids. Therefore, the upregulated expression of genes involved in photosynthesis and glycolysis as well as genes belonging to nitrogen assimilation could well explain the enhanced CO_2_ fixation and protein synthesis in repeated fed-batch culture compared to the batch culture at the molecular level.

### 3.5 Proposed Regulation for the Carbon and Nitrogen Metabolic Pathway in Repeated Fed-Batch Culture

**FIGURE 5:**
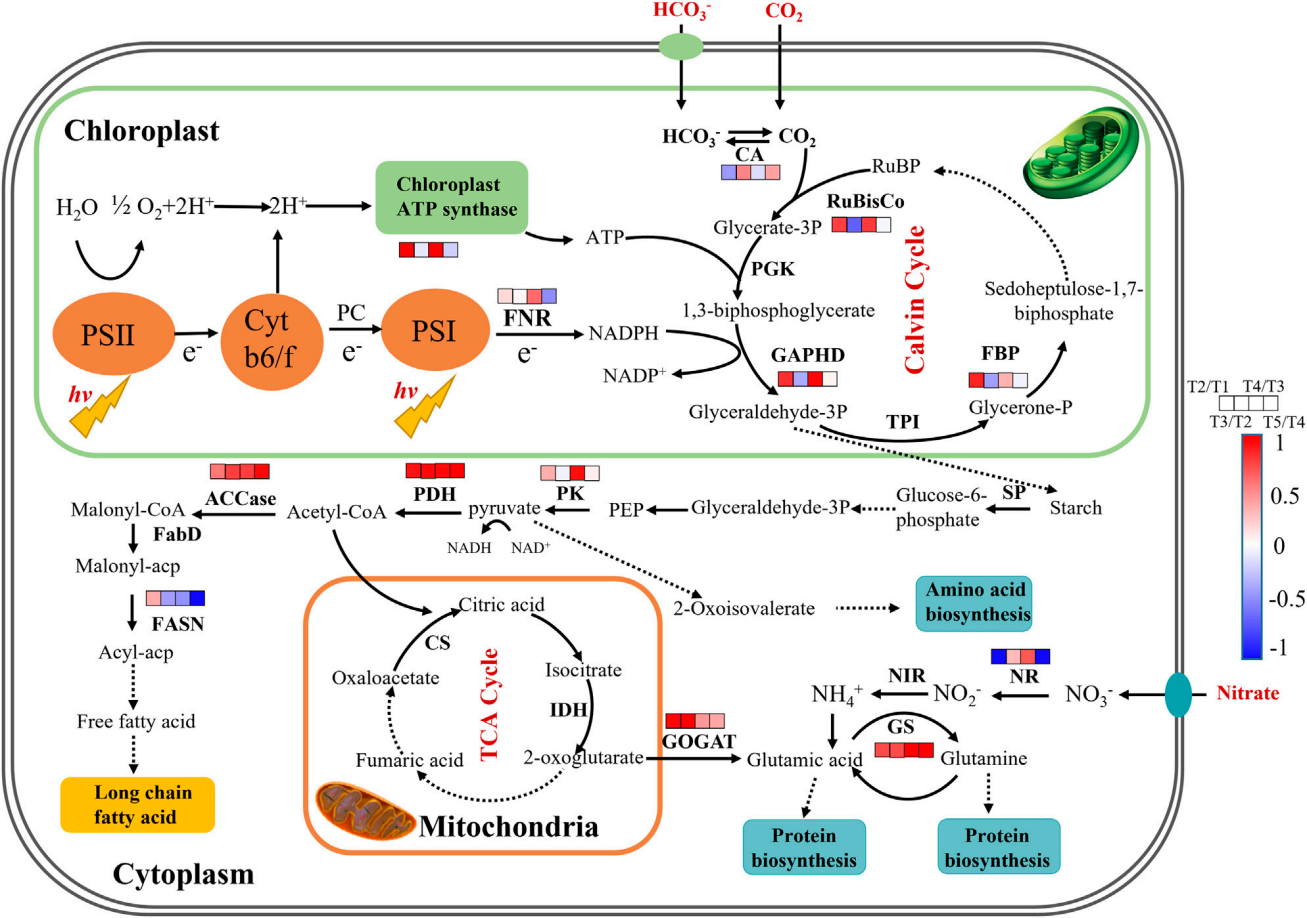
The proposed pathways of enhanced CO_2_ fixation and protein biosynthesis in autotrophic *C. subellipsoidea* during repeated fed-batch culture in a 5-L photo-fermenter. The changes in expression levels of key genes are presented at the time points of 48, 120, 144, 192, and 288 h as T1, T2, T3 and T4, and T5, respectively. Red boxes represent the upregulation of key genes, blue boxes represent the downregulation of key genes, and white boxes represent no significant change in expression of key genes. Solid arrow lines represent the direct reactions between the metabolites, and dash arrow lines represent the multistep reactions among those metabolites.

To reveal the time-dependent regulation of key genes’ expression involved in carbon and nitrogen metabolism, the fresh cells were collected at 48 h (T1), 120 h (T2), and 144 h (T3) in batch 1, and 192 h (T4) and 288 h (T5) in batch 2 of repeated fed-batch culture in 5-L photo-fermenter for qRT-PCR analysis, and the expression level of the key genes was shown in Supplementary Table S3. As shown in Figure 5, most of the key genes in phosphorylation and Calvin cycle were upregulated from T1 to T2 and downregulated or kept stable from T2 to T3 at batch 1; next, they were significantly upregulated again at T4 during the exponential phase in batch 2 and then returned to the initial level or even lower at T5. The upregulated key genes from T3 to T4 related to photophosphorylation, such as *fd* and *alpha-atp,* indicated the enhanced electron transportation due to more available photons harvested by chlorophylls embedded in the thylakoid membrane at batch 2 with weakened light-shading. Meanwhile, the genes encoding RuBisCo, GAPDH, and FBP related to the Calvin cycle were significantly upregulated and then maintained stable, leading to an increased CO_2_ fixation rate continuously from T2 to T5 (Figure 2B). Inversely, the gene coding CA showed different expression patterns with upregulation at T3 and T5 but downregulation at T2 and T4. This might be attributed to the increased pH value resulted from NO_3_^−^ and HCO_3_^−^ consumption in the medium, leading to more HCO_3_^−^ and CO_3_^2-^ but less CO_2_; thus, the upregulation of *ca* expression could converse HCO_3_^−^ to CO_2_ for the use of RuBisco (Wang et al., 2015). Calvin cycle transformed CO_2_ into C_3_ compounds for glycolysis, consequently, the expression of *pk* might be closely linked with the Calvin cycle; and a similar change trend of *pk* expression was certainly observed. The enhanced glycolysis generated more pyruvate and ATP, and then a dramatical upregulation of *pdh* at all time points might immediately promote more pyruvate converted into acetyl-CoA for the TCA cycle, amino acids, and lipid metabolism (Seo et al., 2020; He et al., 2019). At the physiological and biochemical level, the content of protein was significantly higher than that of lipid under high nitrate in repeated fed-batch culture (Figure 3A), indicating the carbon allocation mainly to protein synthesis instead of lipid accumulation. At the transcriptional level, genes encoding GS and GOGAT were both upregulated from T2 to T5, which explained the enhanced protein synthesis; but the expression of gene encoding NR, the rate-limiting enzyme in nitrogen assimilation, did not maintain an upregulated trend. It was dramatically downregulated at T2 and T5 and only showed an upregulation at T4. In fact, the transcription change of *nr* was exactly coincident with that of nitrate consumption. The massive nitrate consumption, 552.25 mg L^−1^ (batch 1) and 646.65 mg L^−1^ (batch 2), accounting for over 30% of total nitrate in the medium, was observed on the first two days in each batch culture, while the slow uptake of nitrate in the later period (Figure 2B). It was reported that the algal cells could take in nitrogen exceeded immediate demand under sufficient nitrogen, allowing organisms to build an “intracellular nitrogen pool” for use in times of scarcity. The nitrogen regulation strategy is called “luxury consumption” in nitrogen assimilation of algae (Bugbee, 2014). The sufficient nitrate transported into the cell induced the high expression level of *nr*, reducing nitrate to nitrite at T1 and T4; and the downregulated expression of *nr* corresponded to slow uptake of nitrate at T2, T3, and T5. In terms of lipid synthesis, the expression of gene coding FASN (rate-limiting enzyme) was sharply downregulated from T3 to T5, resulting in the restriction of fatty acid biosynthesis, even though the expression of *acc* was significantly upregulated. In summary, through analyzing the expression of key genes involved in central carbon and nitrogen metabolism, the underlying molecular mechanisms for regulation of CO_2_ fixation and allocation of carbon flow were primarily elaborated. These achievements would be meaningful for an in-depth understanding of other microalgal responses to different cultivation modes under relative high-level nitrogen.

## 4 Conclusion

In this study, a promising approach of hybrid chemical absorption–biofixation of CO_2_ was developed to enhance CO_2_ fixation and biomass production. Repeated fed-batch culture in a 5-L photo-fermenter resulted in high-protein biomass production, and then, the analysis of key gene expression involved in carbon and nitrogen metabolism revealed the regulation of carbon allocation under high-efficient CO_2_ fixation. The present results proved that *C. subellipsoide* is a promising cell factory for high-efficient CO_2_ fixation with high-protein biomass coproduction, thus expanding the application of this oleaginous microalga in carbon emission reduction and protein production instead of oil production for green manufacturing*.*

## Data Availability

The original contributions presented in the study are included in the article/Supplementary Material; further inquiries can be directed to the corresponding author.

## Abbreviations

CCU, carbon capture and utilization; NR, nitrate reductase; NIR, nitrite reductase; GS/GOGAT, glutamine synthetase/glutamine oxoglutarate aminotransferase or glutamate synthase cycle; DIC, dissolved inorganic carbon; DW, biomass dry weight; R_CO2_, CO_2_ fixation rate; and DLU, degree of lipid unsaturation.
