# Supplementary material for: Oleaginous Microalga Coccomyxa subellipsoidea as a Highly Effective Cell Factory for CO2 Fixation and High-Protein Biomass Production by Optimal Supply of Inorganic Carbon and Nitrogen
Source: Front Bioeng Biotechnol. 2022 Jun 6;10:921024. doi: 10.3389/fbioe.2022.921024 (PMC9207446; doi:10.3389/fbioe.2022.921024)
Supplement: Supplementary file 1 [file DataSheet1.docx]

***Supplementary Material***

# Supplementary Tables

**Table S1** Quality control assessment of the extracted RNA from autotrophic *C. subellipsoidea* in the batch and repeated fed-batch cultures in 5-L photo-fermenters.

| Sample | mRNA concentration (μg mL^-1^) | OD_260nm_ / OD_280nm_ | OD_260nm_ / OD_230nm_ |
| --- | --- | --- | --- |
| **Batch culture** | |  |  |
| 192 h | 167.6±5.68 | 1.9±0.05 | 2.10±0.12 |
| **Repeated fed-batch culture** | | | |
| 48 h | 94.4±2.12 | 1.89±0.20 | 1.90±0.17 |
| 120 h | 394.8±10.11 | 1.98±0.32 | 2.10±0.20 |
| 144 h | 590.8±15.67 | 2.11±0.12 | 1.83±0.03 |
| 192 h | 570.8±29.00 | 2.05±0.18 | 1.89±0.09 |
| 288 h | 175.2±3.27 | 1.90±0.16 | 1.97±0.01 |

Note: the value of OD_260nm_ / OD_280nm_ and OD_260nm_ / OD_230nm_ is between 1.80 and 2.1, which can fit the quality of qRT-PCR requirement.

| **Genes** | **Primer sequence (5’-3’)** | **Genes** | **Primer sequence (5’-3’)** |
| --- | --- | --- | --- |
| *60S ribosomal protein L5* | F: CACGTCTGAGGCTTACTACCC | *pdh* | F: TGAGGAGTGCTTTGACGACC |
|  | R: TCTCCAGCAACAGTGGCATA |  | R: TCCAGGTTGGCAGCATAAGG |
| *fd* | F: TACCCCGCTGTCTTCTGTTG | *acc* | F: TGTCCCCACAGACTGCTCAT |
|  | R: GACCTTGTCTGCTTCATC |  | R: CAGGGTTGGTCTTCTTGCTC |
| *alpha-atp* | F: CACTGCCAAGAGCGAGAAGA | *fasn* | F: AAGGAGTATGGGATTGTGCC |
|  | R: AGCCTATCCACCTGGGAGTC |  | R: TGTGGTAGGCGATGAGGATG |
| *ca* | F: TGGGCGTCTTGTTTACCCTC | *fata* | F: TCTTTGCTGTGACCAGAATGC |
|  | R: AGTATGAGGTGTTGCTCGGC |  | R: GATGATGATCCAGTTGCGG |
| *rbcl* | F: GGAGCCCGTTGACAACAAGT | *nr* | F: GGATCTGCTGAGGACGGATG |
|  | R: CGTTCGAGAACTCCAGAGCA |  | R: GTTCCAGGTGAAGGAGTCCG |
| *gapdh* | F: GACAAGGCCAACTCACACATAAAG | *gs* | F: GCCAGTTCTTGTCTCTGCCT |
|  | R: CGTCGTACTTCTCCTCGTTCA |  | R: AGCTCCACATTGGCACATCA |
|  | F: AACCTCAACGCCATGAACCA | *gogat* | F: AGTCGCTGTTGGTGAGTCTG |
|  | R: CTTGAGCACGGAGTTCTGGA |  | R: GTTGAGGATAGAGCGTGCGA |
| *pk* | F: CAGACGGTGCAGACCATTCT |  |  |
|  | R: ACATGAGGTGCTCGAAGTGG |  |  |

**Table S2** Primer sequence of key genes involved in the central carbon and nitrogen metabolism in autotrophic *C. subellipsoidea.*

**Table S3** The relative expression of key genes involved in the carbon and nitrogen metabolism in repeated fed-batch culture of autotrophic *C. subellipsoidea* in 5-L photo-fermenter.

| **Genes** | **Log_2_ fold change in batch 1** | |  | **Log_2_ fold change in batch 2** | |
| --- | --- | --- | --- | --- | --- |
|  | **T2 / T1** | **T3 / T2** |  | **T4 / T3** | **T5 / T4** |
| *fd* | 1.16±0.03 | -0.1±0.01 |  | 1.78±0.02 | -0.20±0.00 |
| *aphla-atp* | 0.15±0.00 | 0.03±0.00 |  | 0.58±0.01 | -0.47±0.01 |
| *ca* | -0.43±0.02 | 0.48±0.01 |  | -0.15±0.00 | 0.36±0.00 |
| *rcbl* | 0.73±0.03 | -0.66±0.02 |  | 0.76±0.01 | -0.03±0.00 |
| *gapdh* | 0.79±0.01 | -0.35±0.01 |  | 0.93±0.02 | 0.04±0.01 |
| *fbp* | 0.84±0.03 | -0.40±0.01 |  | 0.30±0.00 | -0.06±0.00 |
| *pk* | 0.32±0.00 | -0.06±0.00 |  | 0.91±0.02 | 0.07±0.00 |
| *pdh* | 0.90±0.01 | 0.93±0.02 |  | 0.93±0.01 | 1.74±0.05 |
| *acc* | 0.53±0.02 | 0.73±0.03 |  | 0.75±0.04 | 0.93±0.00 |
| *fasn* | 0.33±0.01 | 0.40±0.01 |  | -0.46±0.00 | -3.70±0.01 |
| *nr* | -1.37±0.04 | 0.28±0.01 |  | 0.65±0.03 | -1.99±0.05 |
| *gs* | 0.70±0.00 | 0.70±0.00 |  | 1.77±0.01 | 1.07±0.02 |
| *gogat* | 0.93±0.00 | 1.04±0.03 |  | 0.40±0.02 | 0.34±0.00 |

Note：The value > 0 means the upregulated expression of key genes, while the value < 0 means the downregulated expression of key genes; T1, T2, T3, T4 and T5 represent the time points of 48, 120, 144, 192 and 288 h in repeated fed-batch culture, respectively.
